# Minimally Invasive Strabismus Surgery: A Systematic Review and Meta-Analysis of Clinical Complications and Efficacy

**DOI:** 10.7759/cureus.90233

**Published:** 2025-08-16

**Authors:** Hosam Hadi Hassan Awaji, Abdullatif H Aljabr, Abdulelah M Alaqel, Rolin T Gharbi, Shahad Mansour Alharthi, Razan Al-Maalwi, Khalid Alsahafi, Alwaleed A Alqahtani, Deema A Alrehaili, Thikra A Alsenani, Bassam A Alzahrani, Raghad Muteb Alruwaili, Sarah M Alsharif

**Affiliations:** 1 Pediatric Medicine, King Salman Armed Forces Hospital (KSAFH), Tabuk, SAU; 2 Medicine, Arabian Gulf University, Riffa, BHR; 3 General Medicine, Prince Mutaib Bin Abdulaziz Hospital, Sakaka, SAU; 4 Faculty of Medicine, Ibnsina College, Jeddah, SAU; 5 Faculty of Medicine, Taif University, Taif, SAU; 6 College of Medicine, King Khalid University, Abha, SAU; 7 Medicine, King Abdulaziz University Hospital, Jeddah, SAU; 8 Medicine, Imam Mohammed ibn Saud Islamic University (IMSIU), Riyadh, SAU; 9 Ophthalmology, Taibah University, Medina, SAU; 10 College of Medicine, King Saud bin Abdulaziz University for Health Sciences (KSAU-HS), Jeddah, SAU; 11 Emergency, Northern Border Health Cluster, Arar, SAU; 12 Ophthalmology, Taif University, Taif, SAU

**Keywords:** complications, conventional strabismus surgery, meta-analysis, minimally invasive surgery, strabismus

## Abstract

Minimally invasive strabismus surgery (MISS) has emerged as a promising alternative to traditional strabismus surgery. This meta-analysis aimed to compare the efficacy and safety of MISS with the standard surgical approach. A comprehensive literature search was conducted to identify relevant studies that included patients who complained of strabismus and underwent MISS or traditional surgeries, English studies, and that were conducted from conception until November 16, 2024. Pooled analysis was performed to compare postoperative alignment, visual acuity, surgery duration, and various postoperative complications between MISS and the standard approach. MISS demonstrated comparable outcomes to the standard approach in terms of postoperative alignment and visual acuity. It was associated with significantly shorter operative times. Both techniques had similar postoperative complication profiles, with MISS showing a lower risk of postoperative scarring. However, the risk of bias in some studies and the limited number of studies for certain outcomes raise concerns about the generalizability of the findings. This meta-analysis suggests that MISS is a safe and effective alternative to traditional strabismus surgery. It offers the advantages of shorter operative time and reduced postoperative scarring. Further high-quality randomized controlled trials are needed to confirm these findings and to assess the long-term outcomes of MISS.

## Introduction and background

Strabismus, commonly known as crossed eyes, is a condition where the eyes do not properly align with each other when looking at an object. It can significantly impact a patient’s quality of life, affecting both visual function and appearance. Traditional strabismus surgery, which involves making a conjunctival incision to access the extraocular muscles, has been the standard treatment for correcting this misalignment. However, it is associated with several drawbacks, including postoperative discomfort, longer recovery times, and visible scarring [1].

While conventional strabismus surgery is generally considered safe and effective, it is not without risks. One of the most common complications is postoperative infection, which can occur at the site of the incision. This can lead to redness, swelling, and discomfort, and in severe cases, may require additional treatment with antibiotics or even further surgical intervention. Another potential complication is scarring, which can be cosmetically unappealing and may also restrict eye movement if the scar tissue forms around the extraocular muscles. Additionally, some patients may experience a recurrence of strabismus, necessitating further surgery to correct the misalignment [2].

Other less common but more serious complications include globe perforation, where the surgical instrument inadvertently penetrates the eye, potentially leading to vision loss. There is also a risk of anterior segment ischemia, a condition where the blood supply to the front part of the eye is compromised, which can result in significant visual impairment. Furthermore, patients may develop diplopia, or double vision, following surgery, which can be temporary or permanent. This can occur if the eyes are over- or under-corrected, or if there is a disruption in the normal binocular vision pathways. These complications highlight the need for careful surgical planning and technique, as well as thorough patient counseling regarding the potential risks and benefits of the procedure [2].

In recent years, minimally invasive strabismus surgery (MISS) has emerged as a promising alternative to conventional techniques. MISS aims to reduce the surgical footprint by utilizing smaller incisions and less disruptive approaches to the extraocular muscles. This technique is designed to minimize tissue damage, potentially leading to reduced postoperative pain, faster recovery, and improved cosmetic outcomes [3].

The purpose of this meta-analysis is to systematically review and synthesize the existing evidence on the efficacy and safety of MISS compared to traditional strabismus surgery. By analyzing data from multiple studies, we aim to provide a comprehensive assessment of the benefits and risks associated with MISS and determine whether it offers a significant advantage over the conventional approach.

## Review

Methodology

Under the registration number CRD420251002956, the protocol for this meta-analysis was submitted to the International Prospective Register of Systematic Reviews (PROSPERO). The Preferred Reporting Items for Systematic Reviews and Meta-Analyses (PRISMA) standards and the Cochrane Handbook for Systematic Reviews of Interventions (version 6) were followed in the conduct and reporting of this meta-analysis [4].

Research aims and objectives

The purpose of this meta-analysis was to evaluate the safety profile of MISS, highlighting the frequency of complications following surgery.

The objectives of the meta-analysis were to systematically review and synthesize existing literature on the outcomes of MISS.

Eligibility criteria

We considered well-conducted, non-randomized, comparative studies (prospective or retrospective cohort studies) and randomized controlled trials (RCTs) published in English from inception to November 16, 2024. The lack of high-quality RCTs on MISS led to the adoption of this strategy. Studies that met the eligibility requirements had to report on at least one of the predetermined outcomes and compare MISS with traditional strabismus surgery. Patients' age, sex, or race were not restricted. Exclusion criteria included duplicate entries, case series with fewer than four patients, conference abstracts, case reports, review articles, and animal research.

Search strategy

The electronic databases Web of Science, ProQuest, Scopus, MEDLINE/PubMed, and the Cochrane Central Register of Controlled Trials (CENTRAL) were searched to locate pertinent papers. The search encompassed all English-language publications up until November 16, 2024. "Strabismus" OR "Ocular Misalignment" AND "Minimally Invasive Strabismus Surgery" OR "MISS" AND "Conventional Strabismus Surgery" OR "Limbal Approach" were the search terms that were utilized. Filters for publication period and language were not used. The first reviewer searched for additional potentially relevant studies that were not identified by the computerized search in the reference lists of the retrieved publications.

Selection of studies

By examining the titles, abstracts, and full texts of the recovered reports, the first reviewer determined their eligibility. After discussing the discrepancies with a third reviewer, the second reviewer confirmed the studies that were retrieved.

Data extraction

A standardized data sheet with the following details was utilized by the first reviewer to obtain data from the included studies: (a) features of the study (author, year, nation, design, and sample size); (b) patient details (age, sex, and sample size at treatment time); (c) intervention specifics (type, side effects, and follow-up period); and (e) the primary outcomes were postoperative conjunctival swelling, injection scores, total inflammatory scores, and duration of surgery; secondary outcomes included foreign body sensation, chemosis, drop intolerance, postoperative alignment, amount of ocular deviation, postoperative scaring, and postoperative visual acuity. The collected data were examined for clarity and consistency by the second reviewer. Any disagreements were resolved by a third reviewer.

Measured outcomes

Primary outcomes included postoperative alignment, amount of ocular deviation, and the changes in the residual angles. Conjunctival swelling following surgery, injection scores, and total inflammatory scores were all evaluated from 0 (nil), 1 (mild), 2 (moderate), and 3 (severe), with the median of each score determined, as well as the duration of surgery (as the mean and standard deviation) in minutes. Secondary outcomes included foreign body sensation, chemosis, drop intolerance (those scores were graded as 0 = nil; 1 = mild; 2 = moderate; and 3 = severe, and the median of each score was calculated), as well as postoperative scaring and postoperative visual acuity.

Evaluation of the risk of bias

The risk of bias (ROB) of the included studies was assessed. We used the National Institute for Health and Care Excellence's (NICE) criteria for randomized controlled clinical trials for assessment [5].

Synthesis of data

**Figure 1:**
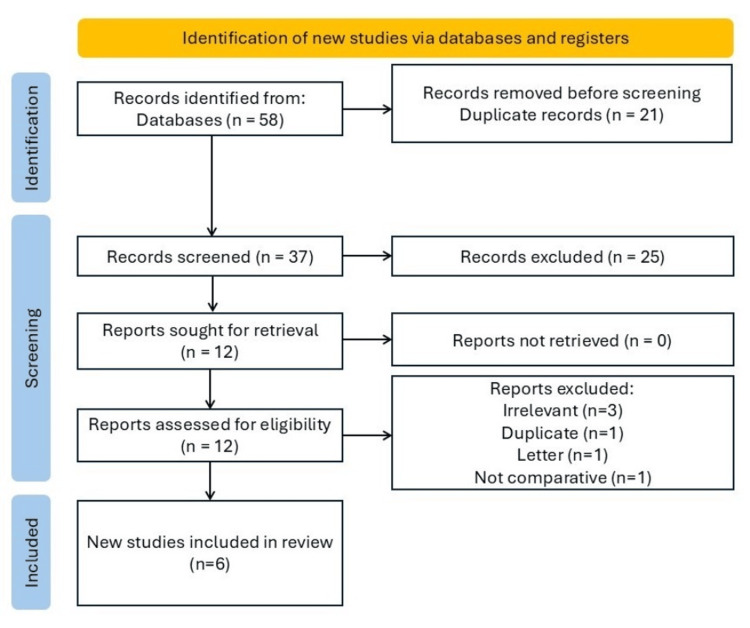
PRISMA flowchart. PRISMA, Preferred Reporting Items for Systematic Reviews and Meta-Analyses

First, searches of electronic databases yielded 58 records. Twelve studies were ultimately deemed eligible after duplicates and excluded studies were eliminated; 6 of these studies (368 eyes) were included (Table 1) [6-11]. The six excluded studies from the MA were either duplicates (*n* = 1), irrelevant (*n *= 3), letters (*n *= 1), or not comparative (*n *= 1), as indicated in Figure 1 [12].

**Table 1:** Summary of the included studies. NM, not mentioned; RCT, randomized controlled trial; M, male; F, female; ∆D, change in diopter power; MISS, minimally invasive strabismus surgery

Author	Year	Country	Study design	Age		Sex		Sample size (number of eyes)		Primary outcomes	Secondary outcomes	Follow-up duration
				MISS	Standard	MISS	Standard	MISS	Standard			
Sharma et al. [6]	2014	India	RCT	14-28 years	14-28 years	9M, 5F	9M, 5F	On 28 eyes of 14 patients, in which one eye was randomized to MISS and the other to SPSS		Duration of surgery	Inflammatory scores, visual acuity, alignment, fusion, stereopsis, residual angle	6 weeks
Pellanda and Mojon [7]	2010	Switzerland	Prospective	2-68 years	NM	25F, 36M	NM	58	132 (48 plication, 84 resection)	Alignment at 6 months (nearness and distance ≤10 pdpt: 50% (28/56), nearness or distance ≤10 pdpt: 73% (41/56))	Binocular vision at 6 months - worsening: 3% (2/56), unchanged: 50% (28/56), improvement: 34% (19/56), not evaluated: 13% (7/56); conjunctival swelling and injection on the first day; dellen, suspected allergy at 6 months	1 day,2 weeks,6 months
Moawad et al. [8]	2021	Egypt	RCT	13.1 ± 10.17 years	9.9 ± 6.45 years	27.9%M, 72.1%F	72.1%M, 27.9%F	15	18	Postoperative ocular alignment (limbal approach: 90.9% success, 9.1% residual angle ˃10 ∆D, MISS: 90.9% success, 9.1% residual angle ˃10 ∆D)	Ocular inflammation: more profound in the limbal approach (*P* < 0.05 for MISS vs. limbal opening plication)	1st day, 1st week, and 1st month
Merino Sanz et al. [9]	2015	Spain	Retrospective	6.75 ± 3.02	(3-12 year) 6.75 ± 3.02	9M, 7F	9M, 7F	16	16	Postoperative deviation	Visual acuity, conjunctival hyperemia, conjunctival swelling, complications	6 months
Mojon [10]	2007	Switzerland	Prospective	27.1 (20.9)	24.4 (20.5)	11M	10M	25	24	Postoperative deviation	Complications, conjunctival redness	6 months
Parveen et al. [11]	2022	India	RCT	21.00 ± 4.98	21.50 ± 6.04	9M, 9F	6M, 12F	18	18	Postoperative conjunctival swelling, injection scores, total inflammatory score, and duration of surgery	Foreign body sensation, chemosis, drop intolerance, postoperative alignment, amount of ocular deviation, postoperative scarring, and visual acuity.	1st day, 2 weeks, 6-8 weeks

Statistical analysis

Review Manager (RevMan) 5.4 was used for the meta-analysis [13]. The amount of ocular alignment and conjunctival chemosis scores was compared using pooled mean differences (MD). Additionally, because of the expected heterogeneity among studies, 95% CIs were computed using a random-effects model, and odds ratios (ORs) were used to compare surgical durations, conjunctival swelling scores, and postoperative alignment among the pooled studies. The I2 statistic was used to measure heterogeneity; values greater than 50% indicated significant heterogeneity. Sensitivity analyses, which eliminated studies one at a time, were conducted to assess the robustness.

Results

A total of six studies involving 368 eyes were conducted to compare the outcomes of MISS with those of the standard surgical approach [6-11].

Primary outcomes

Postoperative Alignment

**Figure 2:**
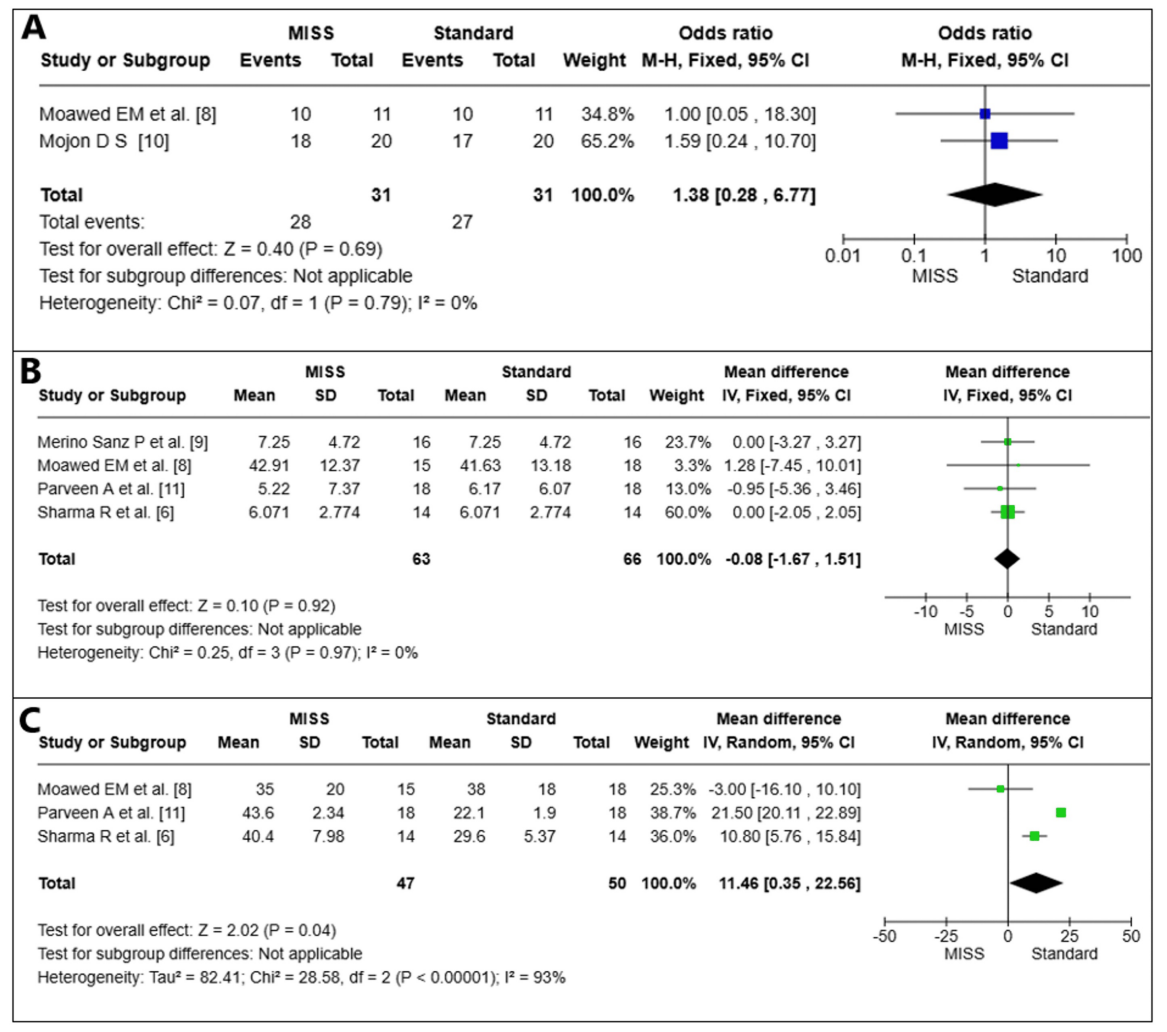
Postoperative alignment (A), degree of ocular deviation (B), and duration of surgery (C). MISS, minimally invasive strabismus surgery; CI, confidence interval

Two pooled studies (*n* = 62 eyes) compared postoperative alignment outcomes between MISS and the standard surgical approach [8,10]. The results of the research showed no statistically significant difference favoring either surgical approach, with an OR of 1.38 (95% confidence interval (CI) 0.28-6.77, *P* = 0.69). With an I2 value of 0%, the heterogeneity analysis revealed little variance among the trials (Figure 2A).

Degree of Postoperative Ocular Deviation

Four pooled studies (*n* = 129 eyes) evaluated the amount of postoperative ocular deviation following MISS compared to the standard surgical approach [6,8,9,11]. The results of the research showed that there was no statistically significant difference in postoperative ocular deviation between the two procedures, with an MD of -0.08 (95% CI -1.67 to 1.51, *P* = 0.92). There was little difference among the trials, according to the heterogeneity analysis, with an I2 score of 0% (Figure 2B).

Surgery Duration

Three pooled studies (*n* = 97 eyes) were examined for the length of the procedure [6,8,11]. The MISS group had a shorter operation duration than the Standard group, with a statistically significant difference (MD = -11.46; 95% CI -22.56 to 0.35, *P *= 0.04). The investigations showed a high degree of heterogeneity (I2 = 93%) that was unresolvable (Figure 2C).

Conjunctival Swelling Score on the First Day Following Surgery

**Figure 3:**
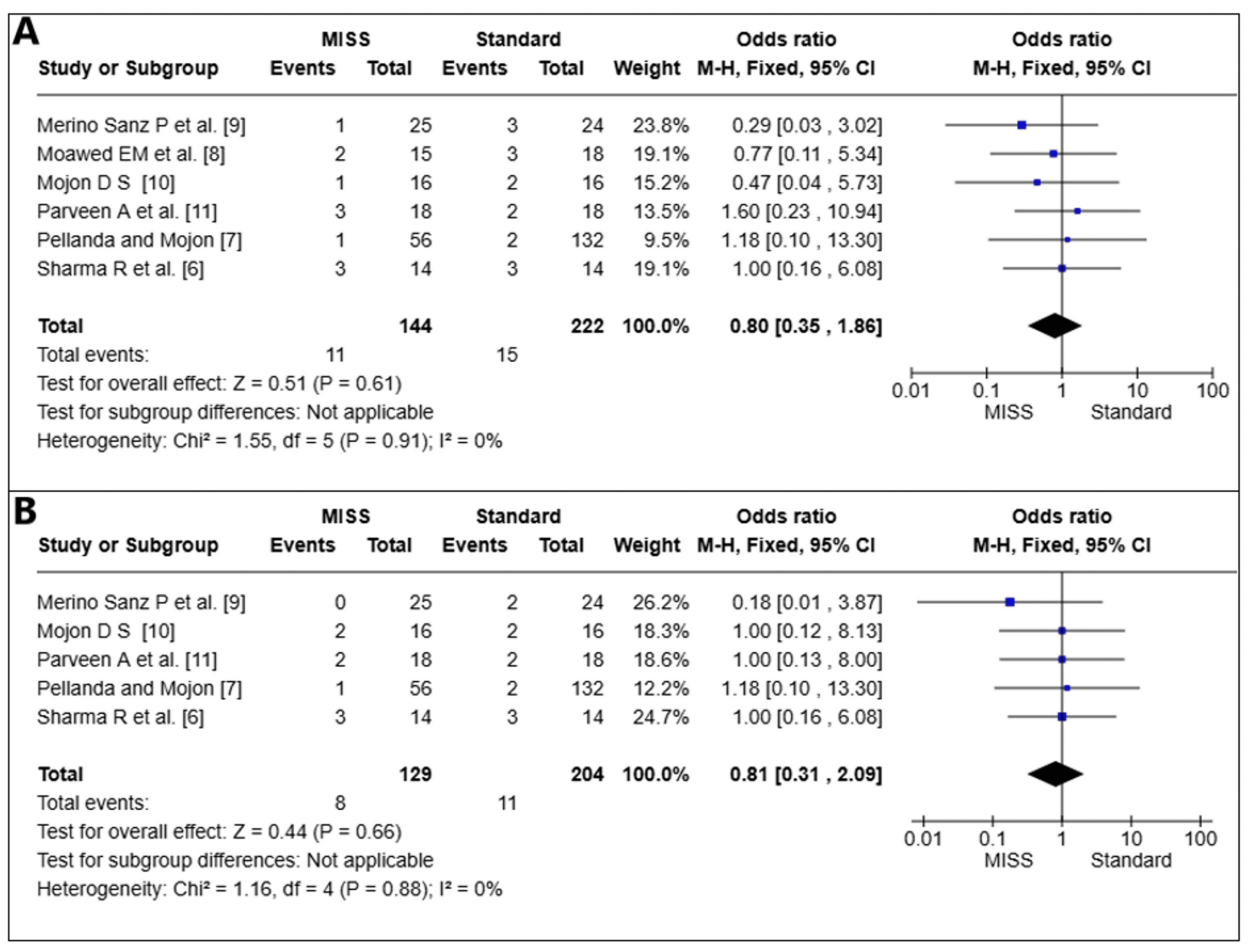
Conjunctival swelling (A) and conjunctival injection (B) scores at the first postoperative day. MISS, minimally invasive strabismus surgery; CI, confidence interval

In six pooled studies (*n* = 366 eyes) [6-11], on the first postoperative day, the conjunctival edema score of (*n *= 366) eyes was examined. The MISS and standard technique did not differ statistically significantly (OR = 0.80; 95% CI 0.35-1.86, *P *= 0.61). There was little variation among the investigations (I2 = 0%) (Figure 3A).

Conjunctival Injection Score on the First Day Following Surgery

In five pooled studies [6,7,9-11], on the first postoperative day, the conjunctival injection score of 3,32 eyes was examined. The MISS and standard methods did not differ statistically significantly (OR = 0.81, 95% CI 0.31-2.09, *P *= 0.66). There was little variation among the investigations (I2 = 0%) (Figure 3B).

Total inflammation score

Total Inflammation Score on the First Postoperative Day

**Figure 4:**
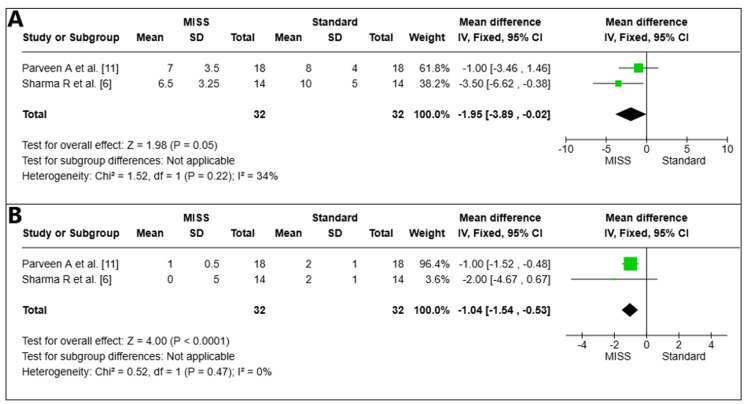
Total inflammation scores on the first day (A) and at the sixth week (B) postoperatively. MISS, minimally invasive strabismus surgery; CI, confidence interval

On the first postoperative day, the overall inflammation score was examined in two pooled studies (*n* = 64 eyes) [6,11]. The MISS group's total inflammatory score was statistically significantly lower than that of the Standard group (MD = -1.95, 95% CI -3.89 to -0.02, *P *= 0.05). Moderate heterogeneity was seen in the studies (I2 = 34%) (Figure 4A).

Total Inflammation Score at Week 6

At week 6 postoperatively, the overall inflammation score of two pooled studies (*n* = 64 eyes) was examined [6,11]. The MISS group had a lower total inflammation score than the Standard group, which was statistically significant (MD = -1.04, 95% CI -1.54 to -0.53, *P *< 0.0001). There was little variation among the investigations (I2 = 0%) (Figure 4B).

Secondary outcomes

Chemosis

**Figure 5:**
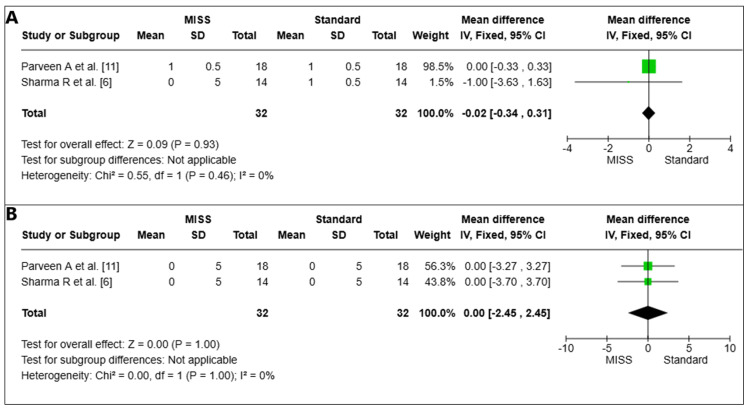
Chemosis on the first day (A) and at the sixth week (B) postoperatively. MISS, minimally invasive strabismus surgery; CI, confidence interval

Chemosis on the first postoperative day: Chemosis was examined in two pooled investigations (*n* = 64 eyes) on the first surgical day [6,11]. The difference between the MISS and Standard groups was not statistically significant (MD = -0.02, 95% CI -0.34 to 0.31, *P *= 0.93). The studies' heterogeneity was minimal (I2 = 0%) (Figure 5A).

Chemosis at week 6 postoperatively: Chemosis was examined in two pooled investigations (*n *= 64 eyes) at week six after surgery [6,11]. The MISS and Standard groups did not differ statistically significantly (MD = 0.00, 95% CI -2.45 to 2.45, *P *= 1.00). There was little variation among the investigations (I2 = 0%) (Figure 5B).

Foreign Body Sensation

**Figure 6:**
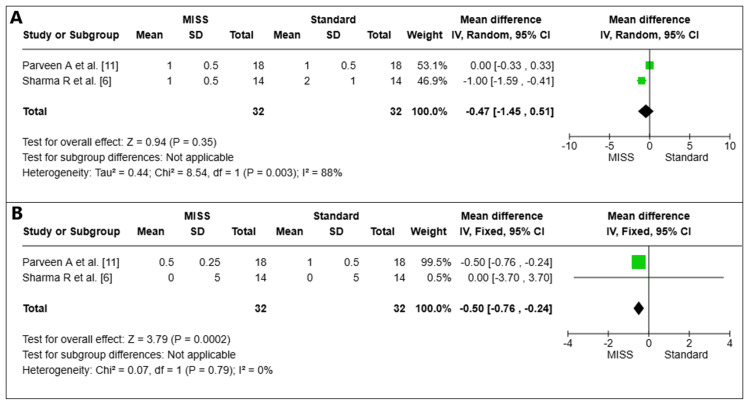
Foreign body sensation on the first day (A) and at the sixth week (B) postoperatively. MISS, minimally invasive strabismus surgery; CI, confidence interval

Foreign body sensation on the first postoperative day: On the first postoperative day, a total of two pooled investigations (*n *= 64 eyes) were examined for foreign body feeling [6,11]. The MISS group experienced less alien body sensation than the Standard group, which was statistically significant (MD = -0.47, 95% CI -1.45 to -0.51, *P *= 0.003). The trials showed significant heterogeneity (I2 = 88%) that was unresolvable (Figure 6A).

Foreign body sensation at week 6 postoperative: At the sixth week after surgery, the foreign body sensation in two pooled trials (*n *= 64 eyes) was examined [6,11]. The MISS group experienced less alien body sensation than the Standard group, which was statistically significant (MD = -0.50, 95% CI -0.76 to -0.24, *P *= 0.0002). There was little variation among the investigations (I2 = 0%) (Figure 6B).

Drop Intolerance

**Figure 7:**
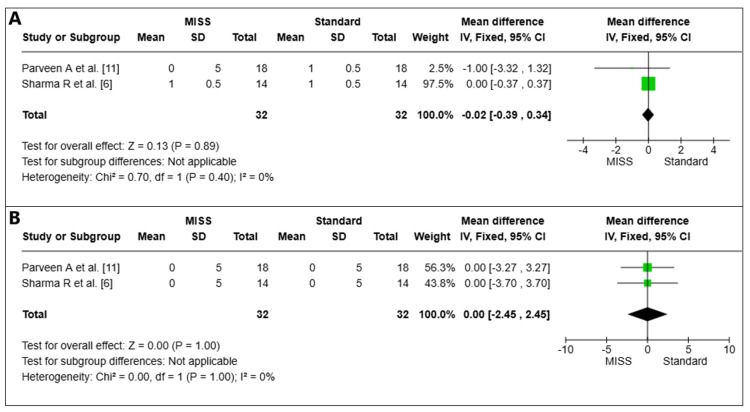
Drop intolerance on the first day (A) and at the sixth week (B) postoperatively. MISS, minimally invasive strabismus surgery; CI, confidence interval

Drop intolerance on the first postoperative day: On the first postoperative day, the drop intolerance of two pooled investigations (*n *= 64 eyes) was examined [6,11]. The MISS and Standard groups did not differ statistically significantly (MD = -0.02, 95% CI -0.39 to 0.34, *P *= 0.89). There was little variation among the investigations (I2=0%) (Figure 7A).

Drop intolerance at week 6: At week six after surgery, drop intolerance was examined in two pooled investigations (*n* = 64 eyes) with a total of 64 eyes [6,11]. The MISS and Standard groups did not differ statistically significantly (MD = 0.00, 95% CI -2.45 to 2.45, *P *= 1.00). There was little variation among the investigations (I2 = 0%) (Figure 7B).

Postoperative Scarring

**Figure 8:**
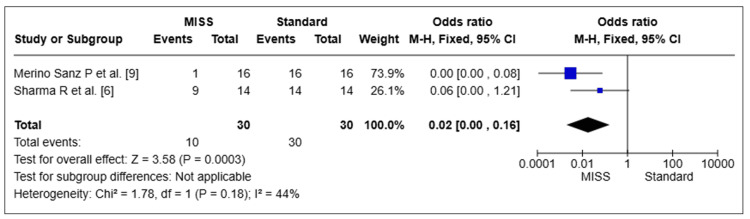
Postoperative scarring. MISS, minimally invasive strabismus surgery; CI, confidence interval

Two pooled studies with a total of 60 eyes were examined for postoperative scarring [6,9]. The MISS group had a decreased incidence of postoperative scarring than the Standard group, which was statistically significant (OR = 0.02, 95% CI 0.00-0.16, *P *= 0.0003). The studies showed moderate heterogeneity (I2 = 44%) (Figure 8).

Postoperative Visual Acuity

**Figure 9:**
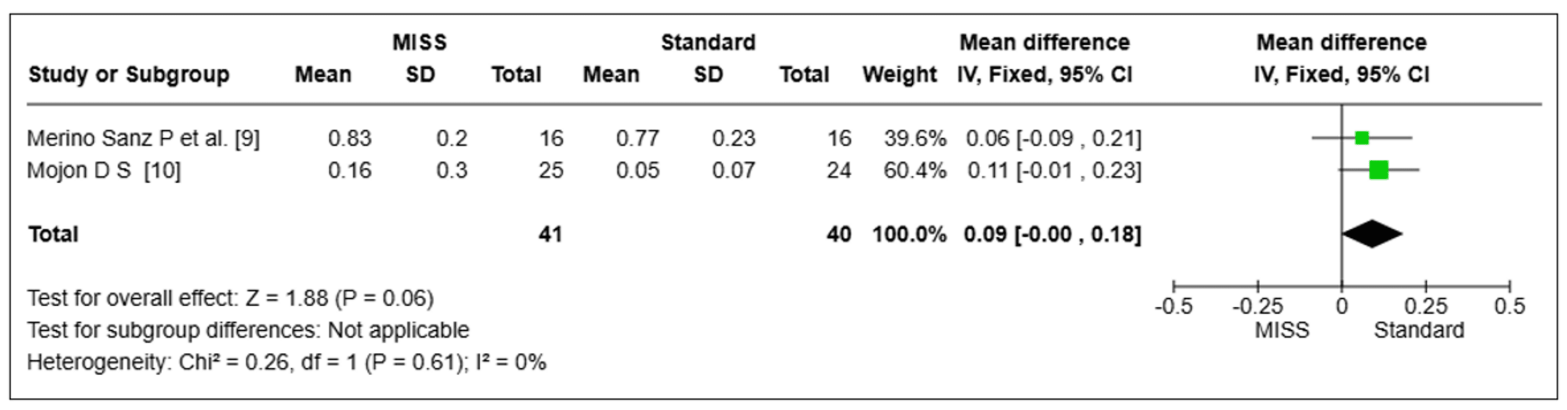
Postoperative visual acuity. MISS, minimally invasive strabismus surgery; CI, confidence interval

The postoperative visual acuity of two pooled investigations (*n* = 81 eyes) was examined [9,10]. The MISS and Standard groups did not differ statistically significantly (MD = 0.09, 95% CI -0.00 to 0.18, *P *= 0.06). There was little variation among the investigations (I2 = 0%) (Figure 9).

Risk of Bias

**Figure 10:**
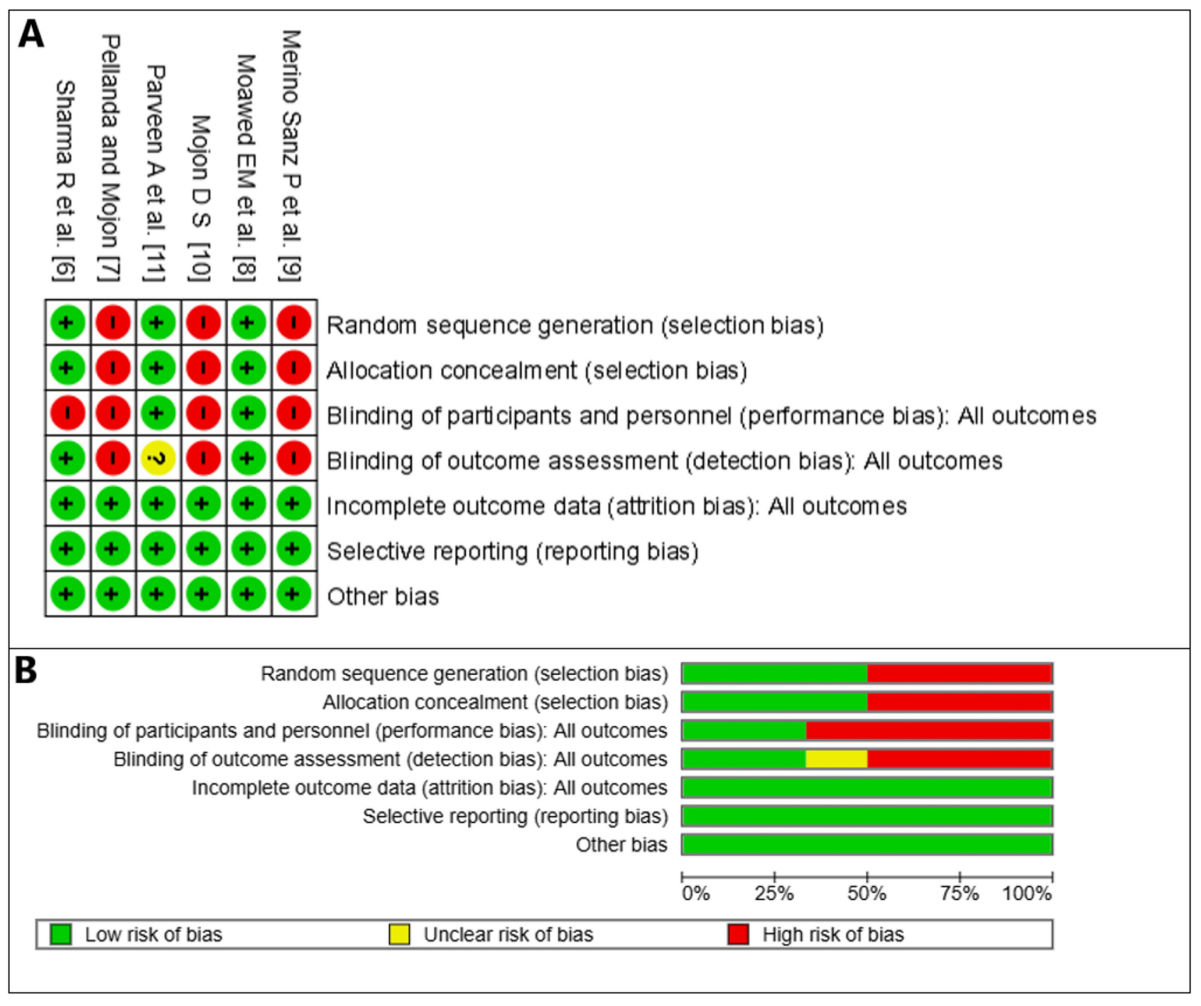
Risk-of-bias summary (A) and graph (B).

Regarding allocation concealment and random generation, half of the studies seem to have a low ROB, as they likely used a method like computer-generated random number sequences or a physical randomization method as well, and the allocation concealment was adequately implemented. However, the randomization and allocation concealment procedures used in the trials by Pellanda and Mojon [7], Merino Sanz et al. [9], and Mojon [10] were not clearly reported, which may have indicated a higher risk of selection bias​​​​​. Blinding of patients and personnel, as well as the assessors, was made in only two studies, which raises the risk of performance and bias detection [6,8]. Every study seems to have a low ROB in the areas of attrition, reporting, or other forms of bias, indicating that the analysis properly considered missing data and that there was little loss to follow-up. Furthermore, studies did not disclose simply positive results; they recorded all relevant outcomes (Figures 10A, 10B).

The high ROB in randomization, allocation concealment, and blinding for several research studies raises questions regarding the validity and reliability of the results, even though the majority of the included studies seem to have a low ROB. The actual treatment impact may be overestimated or underestimated as a result of these biases. When evaluating the meta-analysis's findings, care should be used, especially when it comes to the studies that have a high ROB. To validate these results, more thorough research using a strict methodology is required.

Discussion

Strabismus, a condition characterized by misalignment of the eyes, is commonly treated through surgical intervention. Traditionally, strabismus surgery involves a relatively invasive approach that requires large incisions and extensive tissue dissection. This can lead to postoperative complications such as inflammation, scarring, and visual disturbance [1].

In recent years, MISS has emerged as a promising alternative to traditional techniques. MISS utilizes smaller incisions and less invasive surgical techniques, aiming to reduce postoperative complications and improve patient recovery time [14].

The purpose of this meta-analysis was to evaluate MISS's safety and effectiveness in comparison to the conventional surgical method. According to the study's findings, MISS is a practical and secure substitute for conventional strabismus surgery, with similar results in terms of visual acuity and postoperative alignment.

Regarding primary outcomes, the meta-analysis demonstrated comparable outcomes between MISS and the standard approach in terms of postoperative alignment and ocular deviation. This suggests that MISS can achieve similar levels of precision as traditional surgery.

Concerning secondary outcomes, MISS was associated with significantly shorter operative times compared to the standard approach. This finding suggests that MISS can lead to reduced surgical time and potentially improved efficiency in the operating room.

Regarding postoperative complications, both techniques had similar profiles in terms of conjunctival swelling, injection, chemosis, foreign body sensation, drop intolerance, and total inflammation at both early and late postoperative time points.

One notable advantage of MISS is its association with a significantly lower risk of postoperative scarring, which may lead to improved cosmetic outcomes.

Limitations

Some restrictions should be considered when interpreting these results. The overall results and the findings' ability to be applied broadly may be impacted by the variability seen in certain outcomes, especially the length of surgery and the sensation of a foreign body. Furthermore, the validity of the data raises concerns about the possibility of bias in randomization, allocation concealment, and blinding in certain studies. The results' generalizability is further constrained by the brief follow-up period and the very limited number of studies included for various outcomes.

## Conclusions

MISS is a safe and efficient substitute for the conventional surgical method, according to the meta-analysis's findings. It has the benefits of a quick recovery period and fewer scars after surgery. More rigorous RCTs with strict methodology are necessary to validate these results and evaluate the long-term outcome of MISS.
